# Book Reviews

**Published:** 1908-12

**Authors:** 


### BOOK REVIEWS

GYNECOLOGY AND ABDOMINAL SURGERY. In two large octavos. Edited by Howard A. Kelly, M. D., Professor of Gynecologic Surgery at Johns Hopkins University; and Charles P. Noble, M. D., Clinical Professor of Gynecology at the Woman's Medical College, Philadelphia. Large octavo volume of 862 pages, with 475 original illustrations by Mr. Hermann Becker and Mr. Max Brodel. Philadelphia and London: W. B. Sanders Company, 1908. Per volume: Cloth, $8.00 net; Half Morocco, $9.50 net. W. B. Sanders Company, Philadelphia and London.

The first volume of this splendid work was reviewed sometime ago in our pages and it is again our privilege and pleasure to commend it in the highest possible manner, both as to subject matter and also as regards the excellent illustrations and the high-class of book-making which it so well exemplifies. The limited space forbids a detailed mention of the various subjects covered by the well known surgeons who contributed to this volume; the mere mention of their names is sufficient guarantee of the quality of their contribution, each having taken special interest for years in the subjects discussed. It is with particular pleasure that we note the name of our able and esteemed collaborator Dr. Floyd W. McRae among the authors of Volume II. The full list of authors is as follows:

Brook M. Anspach, M. D.; Joseph C. Bloodgood, B. S., M. D.; John M. T. Finny, A. B., M. D.; Barton Cooke Hirst, A. B., M. D.; Guy L. Hunner, B. S., M. D.; Elizabeth Hurdon, M. D.; George Ben Johnston, M. D.; Howard A. Kelly, A. B., M. D., L.L. D.; F. R. C. S. Hon. Edwin; Edward Martin, A. M., M. D.; Floyd W. McRae, M. D.; G. Brown Miller, B. S., M. D.; B. G. A. Moynihan, M. S., F. R. C. S.; Pohn B. Murphy, A. M., M. D. D. C. L., England, L.L. D.; Charles P. Noble, M.D.S.D.; Richard C. Morris, A. M., M. D.; Albert J. Ochsner, B. S., F. R. M. S., M. D.; Eugene L. Opie, M. D.; J. F. W. Ross, M. D.; Stephen H. Watts, A. M., M. D.; J. Whittridge Williams, A. B., M. D.; S. D.

---

PRINCIPLES OF SURGERY. By Stuart McGuire, M. D., Professor of Principles of Surgery and Clinical Surgery,

University College of Medicine, Richmond, Va. Published by Southern Medical Publishing Co., Baltimore, Md..

The wide experience of Dr. McGuire which he has acquired, added to the natural tendency towards surgery, which he inherited from his distinguished father, makes him peculiarly qualified as a surgeon, while his extensive experience as a teacher have shown him what the student needs. In the above lectures McGuire gives modern views of the principles of surgery in a clear, concise manner that will undoubtedly prove of value to the student and practitioner. The book comprises 480 pages of text from a surgeon; while his extensive experience as a teacher have shown which may be readily obtained a good working knowledge of surgery.

---

DISEASES OF THE NOSE AND THROAT. By C. G. Coakley, A. M., M. D.; 4th edition pp. 604. Published by Lea & Febiger.

The new edition of this manual has been brought up to date. The more recent operations, such as submucous resection of the septum are sufficiently described, and a valuable chapter on therapeutics has been added. The book is worthy of the enviable reputation of its author, and may with confidence be recommended to such as desire a safe guide without unnecessary detail. We have observed, however, not a few printer's errors which somewhat mar the total excellence of the work; for example, on page 120 pulmilionis for pumilions; page 246, figure 52 is not Gruenwald's forceps; page 336, Eucalyptol ℥ss should doubtedly be ʒss. Then we greatly doubt the wisdom of prescribing cocain in hay fever, even with a warning against the danger of becoming habituated to its use, because patients who have once used cocain for any length of time are thereby rendered less susceptible to further improvement by other means. What do the following sentences (page 209) mean? "If pus is seen between the middle turbinate and the septum it is an evidence that either the artrum frontal sinus, or anterior group of eth-modal cells, or some combination of these is involved. If secretion is seen between the middle turbinate and the septum, this indicates involvement of the posterior eth-moidal cells of sphenoidal sinus or both together."

A. W. S.

ARTERIOSCLEROSIS. Etiology, Pathology, Diagnosis Prognosis and Treatment. By Louis M. Warfield, A. B., M. D., instructor in medicine, Washington University, Medical Department; Physician to the Protestant Hospital, Adjunct Attending Physician to the Martha Parson's Hospital for Children, St. Louis, Mo., etc. With introduction by W. S. Thayer, M. D., Professor of Clinical Medicine, Johns Hopkins University. Eight original illustrations. Published by C. V. Mosby Medical Book Co., St. Louis, Mo.

Warfield has summarized the recent ideas and results obtained from experimental research and from clinical observation of arteriosclerosis, and has presented a readable and authoritative monograph which will be of undoubted value to the general practitioner. Stress is appropriately laid on the prevention of arterioclerosis rather than on the treatment of the fully developed disease.

Thayer, in the introduction, urges that care be exercised in making a diagnosis of arterioclerosis and that it should not be used as a cloak for carelessness of ignorance. We heartily commend the work to all of our readers interested in this subject.

---

THE PRACTITIONERS' VISITING LIST FOR 1909. An invaluable pocket-sized book containing memoranda and data important for every physician, and ruled blanks for recording every detail of practice. The Weekly, Monthly and 30-Patient Perpetual contains 32 pages of data and 160 pages of classified blanks. The 60-Patient Perpetual consists of 256 pages of blanks alone. Each in one wallet-shaped book, bound in flexible leather, with flap and pocket, pencil and rubber, and calendar for two years. Price by mail, postpaid, to any address, $1.25. Thumb-letter index, 25 cents extra. Descriptive circular showing the several styles sent on request Lea & Febiger, publishers, Philadelphia and New York.

Being in its twenty-fifth year of issue. The Practitioners' Visiting List embodies the results of long experience and study devoted to its development and perfection.

It is issued in four styles to meet the requirements of every practitioner: "Weekly," dated for 30 patients; "Monthly," undated for 120 patients per month; "Perpetual," undated, for 30 patients weekly per year, and "60 Patients," undated, for 60 patients weekly per year.

The text portion of The Practitioners' Visiting List for 1909 has been thoroughly revised and brought up-to-date. It contains among other valuable information a scheme of dentition; tables of weights and measures and comparative scales; instructions for examining the urine; diagnostic table of eruptive fevers; incompatibles, poisons and antidotes; directions for effecting artificial respiration; extensive table of doses; an alphabetical table of diseases and their remedies, and directions for ligation of arteries. The record portion contains ruled blanks of various kinds, adopted for noting all details of practice and professional business.

---

PRACTICAL POINTS IN ANESTHESIA. By Frederick-Emil Neef, B. S., B. L., M. L., M. D., New York. Price Semi-De Luxe-Cloth 60 cents, post paid. Library. De Luxe Flexible leather $1.50 post paid.
Surgery Publishing Co., 92 William St., N. Y. U. S. A.

This very practical monograph presents the author's impressions on the correct use of chloroform, ether, etc., and is a simple and coherent working method, and is of particular value to those general practitioners who are so situated that the services of a trained anaesthetist cannot be secured. Among the subjects covered are: Induction of Anaesthesia, Cardiac and Respiratory Collapse, When shall the Patient be Declared Ready for Operation, Maintenance of the Surgical Plane of Anaesthesia, Important Reflexes, Vomiting during Anaesthesia, Obstructed Breathing, Use of the Breathing Tube, Indications for Stimulation, Influence of Morphine on Narcosis, General Course of Anaesthesia, Awakening, Recession of Tongue after Narcosis, Post-Operative Distress, Minor Anaesthesia with Ethyl Chloride, Intubation Anaesthesia, etc.

---

A TREATICE ON THE PRINCIPLES AND PRACTICE OF GYNECOLOGY. By E. C. Dudley, A. M., M. D., Professor of Gynecology in the Northwestern University Medical School, Chicago. Fifth edition, thoroughly revised.

Octavo, 806 pages, with 431 illustrations, of which 75 are in colors, and 20 full-page colored plates. Cloth, $5.00, net; leather, $6.00 net; half-morocco, $6.50. Lea & Febiger, publishers, Philadelphia and New York, 1908.

This is the fifth edition of Prof. Dudley's Gynecology within the last ten years. The advantage of presenting this subject according to cause rather than by regions becomes apparent when reading the work of Dudley, who was the first to present gynecology in this manner. The book met with a most cordial reception and, justly so, in its early editions and since then has increased in favor, as well as in excellence by the addition of numerous and illuminating illustrations. It is with pleasure we recommend most highly this book as one of the best of its class. The new edition has been thoroughly revised to date and much new matter and many new illustrations added.

